# Corticosteroid treatment in COVID-19 patients receiving extracorporeal membrane oxygenation: benefit from rational use – authors’ reply

**DOI:** 10.1186/s13613-024-01399-8

**Published:** 2024-10-28

**Authors:** Ryan Ruiyang Ling, Daniel Brodie, Graeme MacLaren, Kollengode Ramanathan

**Affiliations:** 1 Yong Loo Lin School of Medicine, National University of Singapore, National University Health System, Singapore, Singapore; 2 Australia and New Zealand Intensive Care Research Centre, School of Public Health and Preventive Medicine, Monash University, Melbourne, VIC Australia; 3 Department of Anaesthesia, National University Hospital, National University Health System, Singapore, Singapore; 4 Division of Pulmonary and Critical Care Medicine, Department of Medicine, The Johns Hopkins University School of Medicine, Baltimore, MD USA; 5 Cardiothoracic Intensive Care Unit, National University Heart Centre Singapore, National University Health System, Singapore, Singapore

We thank the authors for their interest in our manuscript [1]. In this reply we outline our response to their queries.

We agree that baseline heterogeneity between groups cannot be excluded, in fact, this was highlighted as an important limitation of our study in the published manuscript. The median case volume in our study was 20 VV ECMO cases per year (IQR: 10-34.5). As centre volumes are known to be associated with patient outcomes [2], we had adjusted for centre volumes in the survival model, specifying it as a binary variable (< 30 vs. ≥30 cases per year). The Extracorporeal Life Support Organization (ELSO) does not routinely collect data to derive sequential organ failure assessment (SOFA) score. As in our published manuscript, we refer the readers to the ELSO data dictionaries online. More importantly, studies have shown that prediction scores including SOFA and Respiratory ECMO Survival Prediction (RESP) scores have relatively poorer predictive capacities in patients receiving VV ECMO for COVID-19 [3]. As such, although we agree that such scores are correlated with outcomes, they should not be considered as part of the regression model. Instead, based on prior studies, we adjusted for patient demographics, the year of ECMO initiation, clinical characteristics, and other potential interventions [4, 5]. Systematic reviews have found that the outcomes for ECMO in COVID-19 worsened over time [6, 7], and that the variables we selected were broadly concordant with prognostic factors for mortality in patients with COVID-19 receiving ECMO [8].

We agree that the lack of information on immunomodulatory dosage, duration, and timing makes it challenging to assess their effects. This (again) was acknowledged as an important limitation of our study. We highlighted the fact that immunomodulators (in particular corticosteroids) can be associated with secondary infections. The authors alluded to “patients with low levels of inflammation at baseline”. We believe that our discussion is in alignment with what they propose, i.e. that there is evidence in support of hyperinflammatory and hypoinflammatory phenotypes in ARDS, and these generally correspond to the steroid-responsive and steroid-resistant phenotypes in COVID-19 ARDS [9]. However, this has not been conclusively proven in patients receiving ECMO.

We agree that glucocorticoids, like any other intervention, have potential risks and benefits, which can be altered by how they are used.

## Data Availability

The ELSO data dictionary can be found online at. https://www.elso.org/registry/datadefinitions,forms,instructions.aspx.
